# Zoonotic Soil-Transmitted Helminth Infections among Humans, Gabon

**DOI:** 10.3201/eid3110.250816

**Published:** 2025-10

**Authors:** Huan Zhao, Polydor Ngoy Mutombo, Rodrigue Mintsa-Nguema, Dieudonné Nkoghe, Julienne Atsame, Matthew Watts, Catherine Gordon, Richard S. Bradbury

**Affiliations:** James Cook University, Townsville, Queensland, Australia (H. Zhao, R.S. Bradbury); National Centre for Naturopathic Medicine, Southern Cross University, Lismore, New South Wales, Australia (P.N. Mutombo); Centres for Disease Control and Prevention, Addis Ababa, Ethiopia (P.N. Mutombo); National Centre for Scientific and Technological Research of Gabon, Libreville, Gabon (R. Mintsa-Nguema); Programme National de Lutte contre les Maladies Parasitaires, Ministère de la Santé, Libreville (D. Nkoghe, J. Atsame); Westmead Hospital, Westmead, New South Wales, Australia (M. Watts); University of Sydney, Sydney, New South Wales, Australia (M. Watts); University of Queensland, Brisbane, Queensland, Australia (C. Gordon); QIMR Berghofer Medical Research Institute, Brisbane (C. Gordon)

**Keywords:** Parasites, zoonoses, parasitic diseases, helminths, Necator gorilla, Strongyloides fuelleborni, strongyloidiasis, Ancylostoma, primates, Gabon

## Abstract

We report human infections with *Necator gorillae* and *Strongyloides fuelleborni*, zoonotic helminths from nonhuman primates, in Gabon. We also detected a cryptic *Ancylostoma* species helminth. Infections occurred in settings of localized deforestation and environmental degradation, which increase human–animal contact. Surveillance to clarify the extent of human infections is needed.

Deforestation and environmental degradation across Central Africa, including Gabon, have increasingly brought nonhuman primates (NHPs) close to human settlements. In forest-edge communities, bushmeat hunting and forest resource exploitation further intensify human–NHP interactions (*1*). Those interactions increase the risk for zoonotic parasitic infections from NHP reservoirs to humans, including infections with soil-transmitted helminths (STHs) (*1*,*2*).

In reports from Europe, researchers were infected with *Necator gorillae* hookworm (*3*,*4*) and *Strongyloides fuelleborni fuelleborni* threadworm (*5*) during fieldwork with NHPs in Central Africa, suggesting that zoonotic STH infections might occur extensively in human communities near NHP habitats. A subsequent study among villagers living near NHP habitats in Gabon identified 2 additional human *N. gorillae* infections on the basis of internal transcribed spacer (ITS) 1 and 2 haplotyping analysis (*6*). To determine the extent of zoonotic STH infections in human populations, we conducted a survey of stool samples from villagers in Gabon.

## The Study

We conducted a parasitologic survey of 226 human stool samples in July 2023, during the dry season in Ngounié Province, Gabon. We collected samples from persons in 6 forest-edge communities situated within a tropical savanna climate zone (Köppen classification Aw) (Appendix Figure 1).

**Figure 1:**
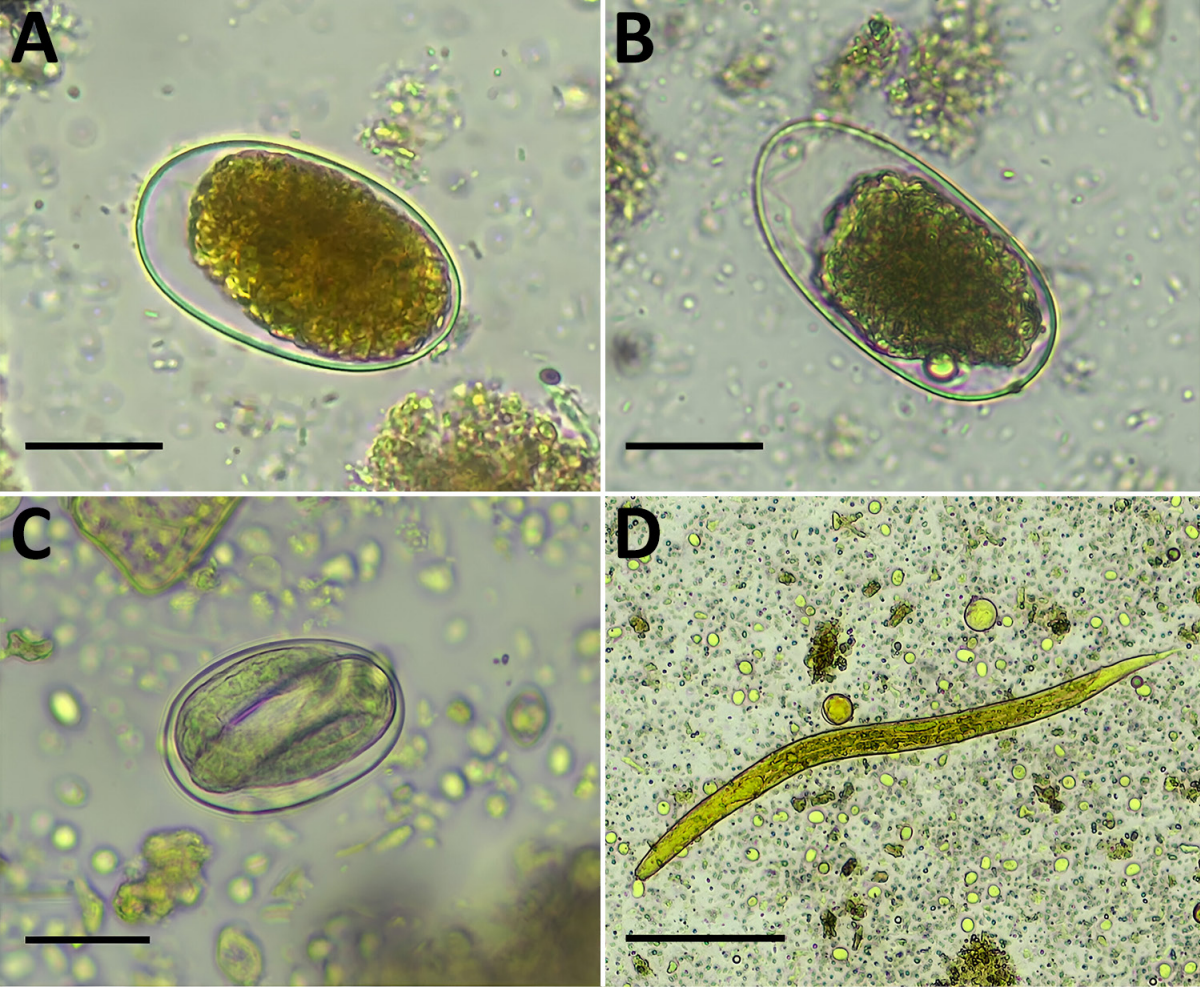
Light microscopy images of soil-transmitted helminths from human stool samples, Gabon. A, B) Hookworm eggs; scale bars indicate 25 µm. C) *Strongyloides* spp. eggs; scale bar indicates 25 µm. D) *Strongyloides* spp. larvae; scale bar indicates 50 µm. Helminths were detected by using the formalin ethyl acetate sedimentation method. Molecular analysis of ethanol-preserved stool sample aliquots from the same patients indicated *Necator americanus* (A), *N. gorillae* (B), *Strongyloides fuelleborni fuelleborni* (C), and *S. stercoralis* (D).

Upon collection, we preserved stool specimens in 10% formalin and 70% ethanol and shipped them to Australia for analysis. Using formalin ethyl acetate sedimentation microscopy (*7*), we identified hookworm eggs in 15 samples, *S. f. fuelleborni* roundworm eggs in 6 samples, and *Strongyloides* spp. roundworm larvae in 1 sample (Figure 1). A total of 20 samples were helminth-positive, and some involved co-infections (Appendix Table 1). 

We performed metabarcoding on DNA extracts from those 20 samples by targeting the mitochondrial cytochrome c oxidase subunit I (*cox1*) gene and the hypervariable region IV (HVR-IV) of 18S rDNA, 2 well-established genetic markers for helminth species identification (*8*). We targeted a 217-bp region of *cox1* to identify helminth species (*8*). Then, to further characterize *Strongyloides* species and genotypes, we conducted a second metabarcoding assay targeting 18S rDNA HVR-IV (≈255 bp) (*8*) on the 7 *Strongyloides* spp.–positive samples. For both assays, we performed sequencing on a MiSeq platform by using MiSeq Reagent Nano Kit v2 (Illumina, https://www.illumina.com) and 500 cycles for 250-bp paired-end reads. We used Geneious Prime version 2024.0.4 (https://www.geneious.com) to analyze sequence data, a custom workflow incorporating read quality control, contig assembly, and haplotype assignment. We conducted phylogenetic analyses of MUSCLE-aligned (https://www.ebi.ac.uk/Tools/msa/muscle) *cox1* sequences by using maximum-likelihood (MEGA 11, https://www.megasoftware.net) and Bayesian inference (MrBayes, https://github.com/NBISweden/MrBayes) methods and applying the general time-reversible nucleotide substitution model. 

Eighteen of the 20 samples yielded *cox1* amplicons. Upon sequencing, 3 samples were dominated by reads from co-infecting *Ascaris lumbricoides* roundworms, but we did not detect sequences for hookworms or *Strongyloides* spp. roundworms. The other 15 samples yielded sequences assigned to *Necator* spp. (n = 11), *S. f. fuelleborni* (n = 3), or *Ancylostoma* spp. (n = 1) helminths (Appendix Table 1). Among the 11 *Necator* spp.–positive samples, 10 harbored *Necator americanus* hookworm, and 4 contained a *Necator* sp. hookworm with *cox1* sequences that had 100% identity to GenBank accession no. AB793562, a species previously detected in researchers from Europe who were infected in the Central African Republic (CAR); that species was later morphologically identified as *N. gorillae* (*3*). Three of the 4 *N. gorillae*–positive samples had *N. americanus* co-infection (Appendix Table 1).

**Figure 2:**
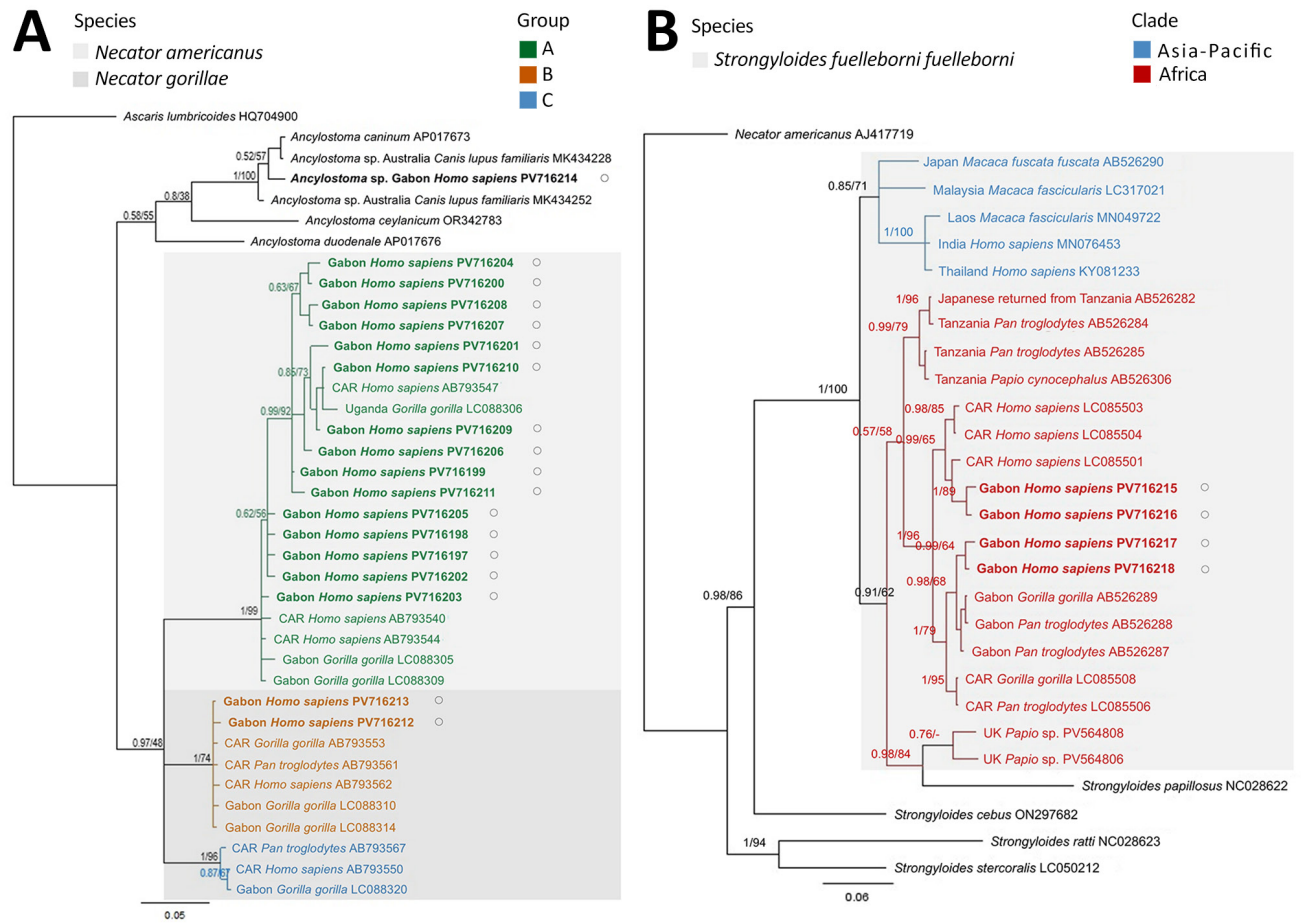
Maximum-likelihood phylogeny of zoonotic soil-transmitted helminths from human infections, Gabon. A) *Necator* spp. hookworms; color-coded groups are labeled per nomenclature by Hasegawa et al. (*4*). B) *Strongyloides fuelleborni fuelleborni* threadworms; color-coded groups represent geographic regions. Trees are based on *cox1* sequences and were created by using MEGA 11 (https://www.megasoftware.net) and Bayesian inference using MrBayes (https://github.com/NBISweden/MrBayes). Bayesian posterior probability and maximum-likelihood bootstrap support percentages (1,000 bootstrap replicates) are indicated at the nodes. Bold font and black circles indicate sequences obtained in this study. Published sequences are annotated with the country of origin, host species, and GenBank accession numbers. Scale bars indicate nucleotide substitutions per site. CAR, Central African Republic

Analysis of *cox1* sequence data revealed 15 haplotypes of *N. americanus* and 2 of *N. gorillae*. Maximum-likelihood and Bayesian inference phylogenetic analyses placed *N. americanus* sequences (217-bp) from this study within a clade containing previously published sequences for that species (Figure 2, panel A). The *N. gorillae* sequences clustered with isolates from NHPs from CAR and Gabon and with isolates recovered from the infected researchers from Europe (Figure 2, panel A). We also detected 1 cryptic *Ancylostoma* sp. hookworm that we could not confidently assign to any known species based on available data. At the *cox1* locus, that *Ancylostoma* sp. hookworm clustered basally to *Ancylostoma caninum* (GenBank accession no. AP017673) and another unidentified *Ancylostoma* sp. hookworm (GenBank accession no. MK434228) identified in dogs from Australia (Figure 2, panel A). We identified 4 *cox1* haplotypes of *S. f. fuelleborni* roundworms, all of which fell within the African clade of that species (Figure 2, panel B). An attempt to sequence the *cox1* of the *S. stercoralis*–positive sample was unsuccessful.

We obtained 18S rDNA HVR-IV sequences of 255–258-bp length from 6 *Strongyloides*-positive samples; we identified 5 as *S. f. fuelleborni* and 1 as *S. stercoralis* (Appendix Table 1). Haplotyping analysis assigned the *S. stercoralis*–positive sample to HVR-IV haplotype A, previously found in humans, dogs, cats, and NHPs (*8*,*9*). *S. f. fuelleborni*–positive samples harbored haplotypes K, L, O, or a combination thereof, previous found in NHPs from Africa and humans (*10*,*11*) (Appendix Figure 2).

## Conclusions

Among sampled communities in Gabon, we found one third (4/12) of hookworm infections were attributable to *N. gorilla*. Multiple *N. gorillae cox1* haplotypes suggest several separate infection events and might be related to higher exposure in certain occupations or other factors, but those data were not available.

Human *Necator* spp. hookworm infections other than *N. americanus* were previously identified on the basis of ITS and *cox1* haplotyping and phylogenetic analysis on samples from 2 researchers returning to Europe from CAR (*4*). Adult worms expelled from 1 researcher were morphologically identified as *N. gorillae* (*3*). Subsequent molecular work in CAR (*12*), Gabon (*6*), and Cameroon (*13*) similarly reported a zoonotic *Necator* sp. hookworm sharing an identical ITS haplotype (II) with those from the researchers from Europe, thus presumably representing *N. gorillae*. Our findings, together with those reports, indicate that *N. gorillae* hookworm infections could be more widespread than currently recognized in certain human communities in Central Africa. Future surveillance for hookworm infections in Central Africa should use species-specific molecular tools to differentiate between human-specific and zoonotic hookworm species.

We do not know whether the novel *Ancylostoma* sp. *cox1* haplotype we identified represents a zoonotic infection from NHPs or another animal, but detection of hookworm eggs in a fecal sample excludes transient passage of ingested DNA. Further investigations using longer read genotyping targets combined with morphologic analysis of harvested adult hookworms could provide more definitive speciation.

Our findings also suggest that *S. f. fuelleborni* roundworm infection is common among human populations in Central Africa. However, little *S. f. fuelleborni* roundworm infection epidemiologic surveillance has been performed in humans in Africa since 1980, when surveys of diagnostic specimens submitted to a hospital in Lusaka, Zambia, reported a 1.0% diagnostic prevalence of *S. f. fuelleborni* roundworm infections over a 7-month period (*14*). A 2024 molecular survey conducted in Asia identified a 3.0% (4/134) infection prevalence in some Bangladesh communities (*15*). 

In our study, *S. f. fuelleborni* sequences clustered closely with isolates from Central Africa at both the *cox1* and 18S rDNA HVR-IV loci, supporting the hypothesis of geographic clustering for this species (*10*,*11*,*15*). The *S. stercoralis* 18S rDNA HVR-IV haplotype we identified is consistent with previous reports of that species in humans from Africa (*8*,*10*).

In summary, we report human infections with *N. gorillae* hookworms and *S. f. fuelleborni* roundworms in Gabon in Central Africa. Those infections occurred in a forest-edge region where localized environmental disturbance and anthropogenic activities, such as hunting and foraging in the adjacent forest, bring villagers into direct contact with NHP habitats, increasing exposure to NHP STHs (*2*). To determine the extent of human infections with zoonotic primate STH in areas where populations overlap and to define the clinical effects and most appropriate treatment strategies for infected persons, enhanced STH surveillance is needed.

Appendix Additional information on zoonotic soil-transmitted helminth infections among humans, Gabon, Central Africa.
