# Supplementary material for: Zoonotic Soil-Transmitted Helminth Infections among Humans, Gabon
Source: Emerg Infect Dis. 2025 Oct;31(10):2029–33. doi: 10.3201/eid3110.250816 (PMC12483102; doi:10.3201/eid3110.250816)
Supplement: Appendix — Additional information on zoonotic soil-transmitted helminth infections among humans, Gabon, Central Africa. [file 25-0816-Techapp-s1.pdf]

Article DOI: https://doi.org/10.3201/eid3110.250816

EID cannot ensure accessibility for supplementary materials supplied by authors. Readers who have difficulty accessing supplementary content should contact the authors for assistance.

Zoonotic Soil-Transmitted Helminth Infections among Humans, Gabon

Appendix

Appendix Table. Species and genotypes of hookworms and *Strongyloides* identified in this study.

			*cox*1		*Strongyloides* 18S HVR-IV	
Sample no.	Microscopy findings	Species assigned	Number of haplotypes	GenBank accession no.	Assigned haplotypes	GenBank accession no.
1	Hookworm eggs	*Necator americanus*	3	PV716197, PV716198, PV716199	–	–
	Strongyloides spp. eggs	*Strongyloides fuelleborni fuelleborni*	–	–	K	PV716221
2	*Strongyloides* larvae	*S. stercoralis*	–	–	A	PV716220
3	*Strongyloides* eggs	NA	NA	NA	–	–
4	*Strongyloides* eggs	*S. f. fuelleborni*	2	PV716215, PV716216	K<br>L<br>O	PV716221,<br>PV716223,<br>PV716222
5	Hookworm eggs; *Strongyloides* eggs	*N. americanus*	2	PV716202, PV716203	–	–
		S. f. fuelleborni	–	–	L	PV716223
6	*Strongyloides* eggs	*S. f. fuelleborni*	1	PV716215	K<br>L<br>O	PV716221,<br>PV716223,<br>PV716222
7	*Strongyloides* eggs	*S. f. fuelleborni*	2	PV716217, PV716218	L<br>O	PV716223,<br>PV716222
8	Hookworm eggs	*Ancylostoma* sp.	1	PV716214	NS	NS
9	Hookworm eggs	*N. americanus*	1	PV716211	NS	NS
10	Hookworm eggs	–	–	–	NS	NS
11	Hookworm eggs	*N. americanus*	1	PV716200	NS	NS
12	Hookworm eggs	*N. americanus*	1	PV716201	NS	NS
13	Hookworm eggs	NA	NA	NA	NS	NS
14	Hookworm eggs	*N. americanus*	1	PV716204	NS	NS
		Necator gorillae	2	PV716212, PV716213†	NS	NS
15	Hookworm eggs	*N. gorillae*	1	PV716212	NS	NS
16	Hookworm eggs	*N. americanus*	1	PV716205	NS	NS
17	Hookworm eggs	NA	NA	NA	NS	NS
18	Hookworm eggs	*N. americanus*	1	PV716206	NS	NS
		N. gorillae	2	PV716212, PV716213†	NS	NS
19	Hookworm eggs	*N. americanus*	2	PV716207, PV716208	NS	NS
		N. gorillae	1	PV716212	NS	NS
20	Hookworm eggs	*N. americanus*	2	PV716209, PV716210‡	NS	NS

*NA, Sequence data showed a dominant presence of *Ascaris* spp., with no detectable hookworm or *Strongyloides* sequences; NS, no sequencing performed; –, sequence not obtained due to amplification failure, sequencing failure, or both.
†Sequence 100% identical to GenBank accession nos. AB793553, AB793554, and AB793562.
‡Sequence 100% identical to GenBank accession no. AB793547.

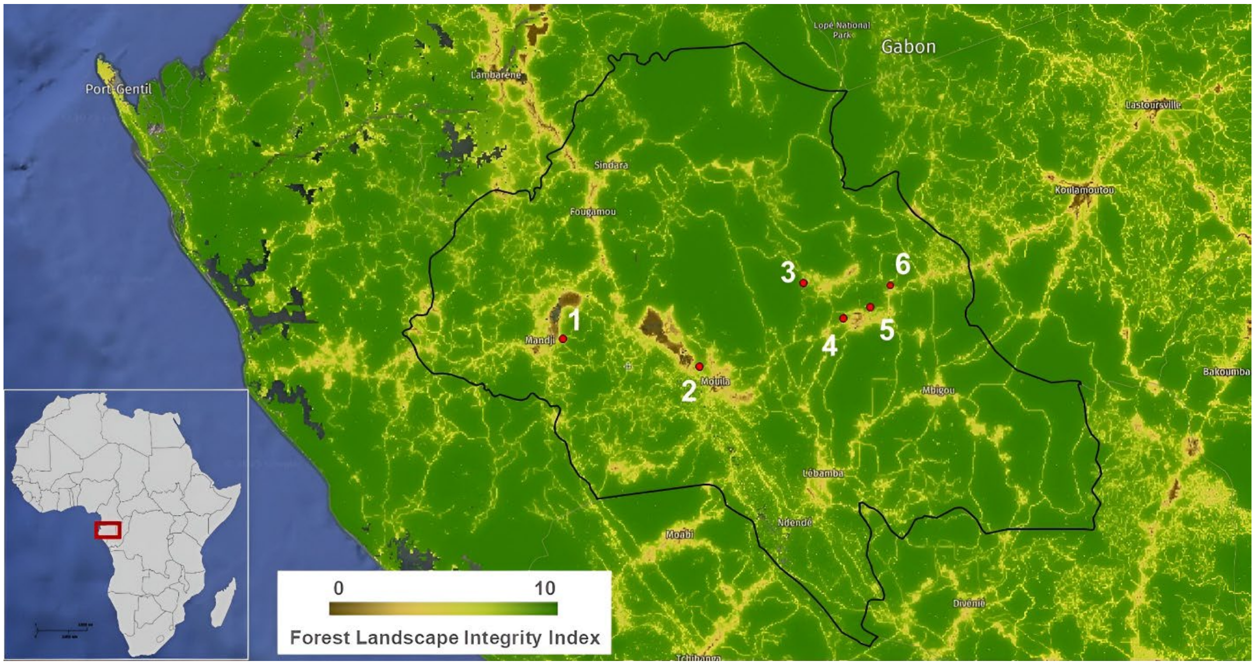

Appendix Figure 1. Heat map of Forest Landscape Integrity Index (FLII) within Gabon, highlighting six study sites: 1. Mandji, 2. Mouila, 3. Eteke, 4. Mimongo, 5. Moukabou, and 6. Dibandi. Dark green areas represent regions with high forest integrity, while yellow and lighter shades indicate areas impacted by human activities. The black boundary outlines Ngounié Province. Data for this map was sourced from the Global Forest Watch FLII dataset (https://www.globalforestwatch.org).

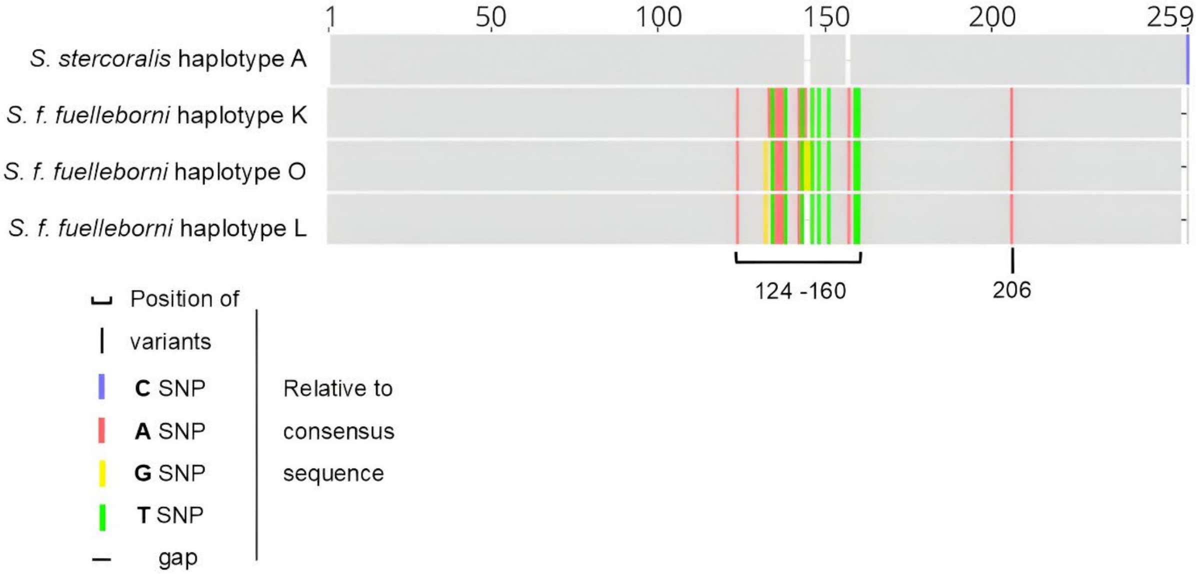

Appendix Figure 2. Schematic of MUSCLE-aligned *18S rRNA* HVR-IV sequences for *Strongyloides fuelleborni fuelleborni* and *Strongyloides stercoralis* detected in human stool samples from Gabon. Haplotypes are indicated as per the nomenclature by Jaleta et al. (*1*) for *S. stercoralis*, and by Barratt et al. (*2*) and Richins et al. (*3*) for *S. f. fuelleborni.*

References

1. Jaleta TG, Zhou S, Bemm FM, Schär F, Khieu V, Muth S, et al. Different but overlapping populations of *Strongyloides stercoralis* in dogs and humans—dogs as a possible source for zoonotic strongyloidiasis. PLoS Negl Trop Dis. 2017;11:e0005752. PubMed https://doi.org/10.1371/journal.pntd.0005752

2. Barratt JLN, Lane M, Talundzic E, Richins T, Robertson G, Formenti F, et al. A global genotyping survey of *Strongyloides stercoralis* and *Strongyloides fuelleborni* using deep amplicon sequencing. PLoS Negl Trop Dis. 2019;13:e0007609. PubMed https://doi.org/10.1371/journal.pntd.0007609

3. Richins T, Sapp SGH, Ketzis JK, Willingham AL, Mukaratirwa S, Qvarnstrom Y, et al. Genetic characterization of *Strongyloides fuelleborni* infecting free-roaming African vervets (*Chlorocebus aethiops sabaeus*) on the Caribbean island of St. Kitts. Int J Parasitol Parasites Wildl. 2023;20:153–61. PubMed https://doi.org/10.1016/j.ijppaw.2023.02.003
